# (*E*)-1-{2-Hy­droxy-5-[(4-methyl­phen­yl)diazen­yl]phen­yl}ethanone

**DOI:** 10.1107/S1600536811001802

**Published:** 2011-01-15

**Authors:** Yasemin Çapan, Canan Kazak, Ayşen Ağar, Mustafa Macit

**Affiliations:** a Ondokuz Mayıs University, Arts and Sciences Faculty, Department of Physics, 55139 Samsun, Turkey; b Ondokuz Mayıs University, Arts and Sciences Faculty, Department of Chemistry, 55139 Samsun, Turkey

## Abstract

The structure of the title compound, C_15_H_14_N_2_O_2_, an azo dye, displays a *trans* configuration with respect to the N=N bridge. The dihedral angle between the aromatic rings is 5.06 (8)°. The mol­ecular conformation is stabilized by a strong intra­molecular O—H⋯O hydrogen bond. In the crystal, inter­molecular C—H⋯O hydrogen bonds occur.

## Related literature

For general backgrond to azo compounds, see: Klaus (2003 ▶); Bahatti & Seshadri (2004 ▶); Catino & Farris (1985 ▶); Fadda *et al.* (1994 ▶);Taniike *et al.* (1996 ▶); Zollinger (2003 ▶); For bond-length data, see: El-Ghamry *et al.* 2008 ▶; Petek *et al.* 2006 ▶.
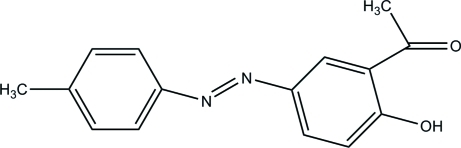


## Experimental

### 

#### Crystal data

C_15_H_14_N_2_O_2_
*M* *_r_* = 254.28
Triclinic, 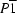

*a* = 7.0919 (7) Å
*b* = 7.0842 (7) Å
*c* = 13.4094 (13) Å
α = 92.722 (8)°
β = 93.045 (8)°
γ = 101.926 (9)°
*V* = 657.04 (11) Å^3^
*Z* = 2
Mo *K*α radiation
μ = 0.09 mm^−1^
*T* = 296 K
0.42 × 0.34 × 0.19 mm

#### Data collection

Stoe IPDS 2 diffractometer
Absorption correction: integration (*X-RED32*; Stoe & Cie, 2002 ▶) *T*_min_ = 0.974, *T*_max_ = 0.991
8340 measured reflections
2542 independent reflections
791 reflections with *I* > 2σ(*I*)
*R*_int_ = 0.090

#### Refinement

*R*[*F* ^2^ > 2σ(*F* ^2^)] = 0.054
*wR*(*F* ^2^) = 0.100
*S* = 0.82
2542 reflections
173 parameters
H-atom parameters constrained
Δρ_max_ = 0.10 e Å^−3^
Δρ_min_ = −0.11 e Å^−3^

### 

Data collection: *X-AREA* (Stoe & Cie, 2002 ▶); cell refinement: *X-AREA*; data reduction: *X-RED32* (Stoe & Cie, 2002 ▶); program(s) used to solve structure: *SHELXS97* (Sheldrick, 2008 ▶); program(s) used to refine structure: *SHELXL97* (Sheldrick, 2008 ▶); molecular graphics: *ORTEP-3 for Windows* (Farrugia, 1997 ▶); software used to prepare material for publication: *WinGX* (Farrugia, 1999 ▶) and *PLATON* (Spek, 2009 ▶).

## Supplementary Material

Crystal structure: contains datablocks global, I. DOI: 10.1107/S1600536811001802/hg2784sup1.cif

Structure factors: contains datablocks I. DOI: 10.1107/S1600536811001802/hg2784Isup2.hkl

Additional supplementary materials:  crystallographic information; 3D view; checkCIF report

## Figures and Tables

**Table 1:** Hydrogen-bond geometry (Å, °)

*D*—H⋯*A*	*D*—H	H⋯*A*	*D*⋯*A*	*D*—H⋯*A*
O1—H16⋯O2	0.82	1.80	2.533 (4)	147
C14—H14*C*⋯O1^i^	0.96	2.53	3.318 (4)	139

Symmetry code: (i) 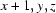.

**Fig. 1.**
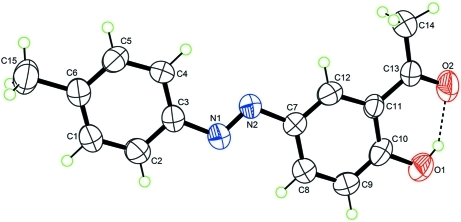
An ORTEP view of (I), with the atom-numbering scheme and 30% probability displacement ellipsoids. Dashed lines indicate H-bonds.

**Fig. 2.**
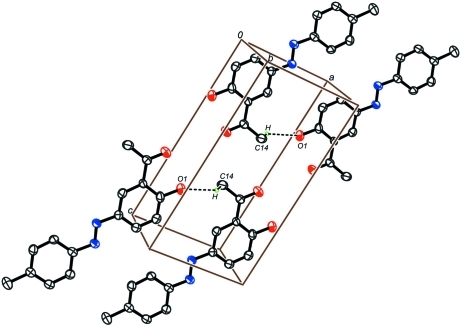
Part of the crystal structure of the title compound, showing the O—H···O and C—H···O interactions. For clarity, only H atoms involved in hydrogen bonding have been included. For symmetry codes, see Table 1.

## supplementary crystallographic information

### Comment

Azo compounds are very important in the field of dyes, pigments and advanced materials (Klaus, 2003). It has been known for many years that the azo compounds are the most widely used class of dyes, due to their versatile applications in various field such as dyeing of textile fibers, the coloring of different meterials, colored plastic and polymers, biological-medical studies and advanced applications in organic synthesis (Bahatti & Seshadri, 2004; Catino & Farris, 1985; Fadda *et al.* 1994; Taniike *et al.* 1996; Zollinger 2003).

In the structure of (I) (Fig. 1) the two aromatic rings are in a *trans* configuration with respect to a azo double bond. The dihedral angel between mean planes of the benzene ring *A*(C1—C6), azo bridge *B* (C3—N1═N2—C7) and other benzene ring *C*(C7—C12)are 2.15 (13)° (*A/C*), 3.64 (10)° (*B/C*), respectively. The N1—C3 [1.431 (4) Å] and N2—C7 [1.425 (4) Å] bond distances are of single bonds character, whereas, N1═N2 (1.251 (2) Å) bond distance is double bond character and compare with literature values of 1.439 (3), 1.428 (2) and 1.248 (2)Å, respectively (Petek *et al.* 2006). All the other bond lenghts are in aggrement with reported for other azo compounds (El-Ghamry *et al.* 2008). Crystals of I were found to be twinned that the twinned cell can be obtained by the unit-celltransformation atwinvector = –avector, btwinvector = bvector, ctwinvector = –cvector, indicating a twofold twinning axis along [010] direction.

The crystal structure is stabilized by one intramolecular O1—H···O2 (Fig. 1, Table 1) and intermolecular C14—H···O1 hydrogen bonds (Fig. 2).

### Experimental

A mixture of *p*-tolidune (10 mmol, 1.07 g) water (10 ml) and conc. HCl (2.8 ml) was stirred until clear solution was obtained. This solution was cooled down to 0–5°C and a solution in water of NaNO_2_ (0.74 g, 15 mmol) was added dropwise and stirred for 1 h below 5°C. The solution of 2-hydroxyacetophenone (1.45 g, 10.7 mmol) was added to a cooled solution of benzenediazoniumchloride and stirred at 0–5 ° C for 1 h. (*E*)-1-(2-hydroxy-4-(*p*-tolydiazenyl)phenylethanone was recrystallized from ethanol (Yield 78%; m.p. = 420–423K).

### Refinement

All H-atoms were refined using a riding model with d(O—H)= 0.82 Å and d(C—H)= 0.96 Å (*U*_iso_ = 1.2 *U*_eq_ of the parent atom) for aromatic C atoms.

The crystal used for the intensity data collection was a non-merohedral twin with two reciprocal lattices differently oriented according to the twofold rotation axis (010), giving rise to double diffraction spot sets. The two data sets of the twin parts were integrated separately and then scaled to give the combined data set. However, because the partially overlapped reflections could not be satisfactorily integrated separately, they were discarded leading to a data completeness of only slightly over 31%

### Crystal data

**Table:**

C_15_H_14_N_2_O_2_	*Z* = 2
*M**_r_* = 254.28	*F*(000) = 268
Triclinic, *P*1	*D*_x_ = 1.285 Mg m^−^^3^
Hall symbol: -P 1	Mo *K*α radiation, λ = 0.71073 Å
*a* = 7.0919 (7) Å	Cell parameters from 4309 reflections
*b* = 7.0842 (7) Å	θ = 2.9–27.7°
*c* = 13.4094 (13) Å	µ = 0.09 mm^−^^1^
α = 92.722 (8)°	*T* = 296 K
β = 93.045 (8)°	Prism, brown
γ = 101.926 (9)°	0.42 × 0.34 × 0.19 mm
*V* = 657.04 (11) Å^3^	

### Data collection

**Table:**

Stoe IPDS 2 diffractometer	2542 independent reflections
Radiation source: fine-focus sealed tube	791 reflections with *I* > 2σ(*I*)
graphite	*R*_int_ = 0.090
Detector resolution: 6.67 pixels mm^-1^	θ_max_ = 27.6°, θ_min_ = 2.9°
rotation method scans	*h* = −9→9
Absorption correction: integration (*X-RED32*; Stoe & Cie, 2002)	*k* = −9→9
*T*_min_ = 0.974, *T*_max_ = 0.991	*l* = −17→17
8340 measured reflections	

### Refinement

**Table:**

Refinement on *F*^2^	Primary atom site location: structure-invariant direct methods
Least-squares matrix: full	Secondary atom site location: difference Fourier map
*R*[*F*^2^ > 2σ(*F*^2^)] = 0.054	Hydrogen site location: inferred from neighbouring sites
*wR*(*F*^2^) = 0.100	H-atom parameters constrained
*S* = 0.82	*w* = 1/[σ^2^(*F*_o_^2^) + (0.0254*P*)^2^] where *P* = (*F*_o_^2^ + 2*F*_c_^2^)/3
2542 reflections	(Δ/σ)_max_ = 0.002
173 parameters	Δρ_max_ = 0.10 e Å^−^^3^
0 restraints	Δρ_min_ = −0.11 e Å^−^^3^

### Special details

**Table:**

Geometry. All e.s.d.'s (except the e.s.d. in the dihedral angle between two l.s. planes) are estimated using the full covariance matrix. The cell e.s.d.'s are taken into account individually in the estimation of e.s.d.'s in distances, angles and torsion angles; correlations between e.s.d.'s in cell parameters are only used when they are defined by crystal symmetry. An approximate (isotropic) treatment of cell e.s.d.'s is used for estimating e.s.d.'s involving l.s. planes.
Refinement. Refinement of *F*^2^ against ALL reflections. The weighted *R*-factor *wR* and goodness of fit *S* are based on *F*^2^, conventional *R*-factors *R* are based on *F*, with *F* set to zero for negative *F*^2^. The threshold expression of *F*^2^ > σ(*F*^2^) is used only for calculating *R*-factors(gt) *etc*. and is not relevant to the choice of reflections for refinement. *R*-factors based on *F*^2^ are statistically about twice as large as those based on *F*, and *R*- factors based on ALL data will be even larger.

### Fractional atomic coordinates and isotropic or equivalent isotropic displacement parameters (Å^2^)

**Table:**

	*x*	*y*	*z*	*U*_iso_*/*U*_eq_	
C1	0.5890 (6)	0.1644 (5)	0.6539 (3)	0.0626 (10)	
H1	0.5339	0.1331	0.5892	0.075*	
C2	0.4713 (5)	0.1621 (5)	0.7323 (3)	0.0613 (10)	
H2	0.3380	0.1268	0.7201	0.074*	
C3	0.5494 (5)	0.2116 (5)	0.8289 (2)	0.0498 (9)	
C4	0.7472 (5)	0.2595 (5)	0.8461 (3)	0.0565 (10)	
H4	0.8021	0.2911	0.9108	0.068*	
C5	0.8635 (5)	0.2602 (5)	0.7668 (3)	0.0615 (10)	
H5	0.9969	0.2939	0.7788	0.074*	
C6	0.7860 (5)	0.2119 (5)	0.6695 (3)	0.0569 (9)	
C7	0.3481 (5)	0.2497 (5)	1.0644 (3)	0.0513 (9)	
C8	0.1491 (5)	0.2170 (5)	1.0434 (3)	0.0637 (10)	
H8	0.0991	0.1991	0.9773	0.076*	
C9	0.0274 (5)	0.2111 (5)	1.1187 (3)	0.0699 (11)	
H9	−0.1053	0.1879	1.1040	0.084*	
C11	0.3008 (5)	0.2802 (4)	1.2409 (2)	0.0455 (9)	
C12	0.4198 (5)	0.2828 (4)	1.1621 (3)	0.0535 (10)	
H12	0.5529	0.3079	1.1759	0.064*	
C13	0.3760 (5)	0.3186 (5)	1.3452 (3)	0.0553 (9)	
C14	0.5883 (5)	0.3741 (5)	1.3698 (3)	0.0698 (10)	
H14A	0.6130	0.3940	1.4410	0.105*	
H14B	0.6403	0.4913	1.3387	0.105*	
H14C	0.6484	0.2728	1.3456	0.105*	
C15	0.9185 (6)	0.2152 (5)	0.5837 (3)	0.0842 (13)	
H15A	1.0252	0.1579	0.6031	0.126*	
H15B	0.9660	0.3464	0.5676	0.126*	
H15C	0.8477	0.1434	0.5262	0.126*	
C10	0.1004 (5)	0.2396 (5)	1.2172 (3)	0.0604 (10)	
N1	0.4117 (4)	0.2099 (4)	0.9032 (2)	0.0546 (8)	
N2	0.4863 (4)	0.2508 (4)	0.9904 (2)	0.0511 (8)	
O1	−0.0261 (3)	0.2310 (3)	1.2899 (2)	0.0836 (8)	
H16	0.0334	0.2494	1.3448	0.125*	
O2	0.2651 (4)	0.3054 (4)	1.41403 (18)	0.0814 (8)	

### Atomic displacement parameters (Å^2^)

**Table:**

	*U*^11^	*U*^22^	*U*^33^	*U*^12^	*U*^13^	*U*^23^
C1	0.066 (3)	0.076 (3)	0.044 (2)	0.015 (2)	0.003 (2)	−0.0022 (18)
C2	0.053 (2)	0.074 (3)	0.055 (2)	0.0093 (19)	0.0023 (19)	0.0057 (19)
C3	0.065 (3)	0.047 (2)	0.041 (2)	0.0192 (19)	0.0056 (19)	0.0092 (17)
C4	0.047 (2)	0.074 (3)	0.049 (2)	0.0155 (19)	−0.0025 (18)	0.0000 (18)
C5	0.049 (2)	0.071 (3)	0.065 (3)	0.0132 (18)	0.008 (2)	0.0025 (19)
C6	0.068 (3)	0.060 (2)	0.045 (2)	0.0181 (19)	0.0076 (19)	0.0055 (17)
C7	0.044 (2)	0.056 (3)	0.053 (2)	0.0100 (18)	0.0088 (18)	0.0016 (19)
C8	0.056 (2)	0.080 (3)	0.054 (2)	0.0128 (19)	0.0020 (19)	−0.0057 (19)
C9	0.043 (2)	0.098 (3)	0.067 (3)	0.0132 (19)	0.002 (2)	−0.006 (2)
C11	0.046 (2)	0.049 (2)	0.043 (2)	0.0108 (18)	0.0100 (18)	0.0006 (16)
C12	0.050 (2)	0.051 (3)	0.060 (3)	0.0096 (18)	0.003 (2)	0.0026 (19)
C13	0.058 (2)	0.059 (2)	0.050 (2)	0.0145 (18)	0.0114 (19)	−0.0050 (16)
C14	0.068 (2)	0.087 (3)	0.053 (2)	0.0194 (19)	−0.0019 (17)	−0.0088 (18)
C15	0.088 (3)	0.108 (3)	0.065 (3)	0.031 (2)	0.031 (2)	0.013 (2)
C10	0.050 (2)	0.065 (3)	0.067 (3)	0.0109 (18)	0.022 (2)	−0.0025 (19)
N1	0.060 (2)	0.062 (2)	0.0427 (18)	0.0132 (15)	0.0040 (15)	0.0017 (15)
N2	0.053 (2)	0.0559 (19)	0.046 (2)	0.0132 (15)	0.0055 (16)	0.0022 (14)
O1	0.0542 (17)	0.119 (2)	0.0733 (18)	0.0082 (14)	0.0229 (14)	−0.0091 (14)
O2	0.078 (2)	0.109 (2)	0.0548 (17)	0.0120 (15)	0.0216 (15)	−0.0074 (14)

### Geometric parameters (Å, °)

**Table:**

C1—C6	1.370 (5)	C9—C10	1.383 (5)
C1—C2	1.375 (5)	C9—H9	0.9300
C1—H1	0.9300	C11—C12	1.385 (5)
C2—C3	1.382 (4)	C11—C10	1.406 (4)
C2—H2	0.9300	C11—C13	1.463 (4)
C3—C4	1.377 (4)	C12—H12	0.9300
C3—N1	1.430 (4)	C13—O2	1.239 (4)
C4—C5	1.380 (5)	C13—C14	1.490 (4)
C4—H4	0.9300	C14—H14A	0.9600
C5—C6	1.386 (4)	C14—H14B	0.9600
C5—H5	0.9300	C14—H14C	0.9600
C6—C15	1.522 (5)	C15—H15A	0.9600
C7—C12	1.371 (5)	C15—H15B	0.9600
C7—C8	1.393 (4)	C15—H15C	0.9600
C7—N2	1.431 (4)	C10—O1	1.354 (4)
C8—C9	1.360 (5)	N1—N2	1.253 (3)
C8—H8	0.9300	O1—H16	0.8200
			
C6—C1—C2	121.1 (4)	C12—C11—C10	117.3 (3)
C6—C1—H1	119.4	C12—C11—C13	122.6 (3)
C2—C1—H1	119.4	C10—C11—C13	120.1 (3)
C1—C2—C3	120.6 (4)	C7—C12—C11	122.2 (4)
C1—C2—H2	119.7	C7—C12—H12	118.9
C3—C2—H2	119.7	C11—C12—H12	118.9
C4—C3—C2	119.1 (4)	O2—C13—C11	120.8 (3)
C4—C3—N1	125.8 (3)	O2—C13—C14	119.2 (3)
C2—C3—N1	115.1 (3)	C11—C13—C14	120.1 (3)
C3—C4—C5	119.7 (4)	C13—C14—H14A	109.5
C3—C4—H4	120.2	C13—C14—H14B	109.5
C5—C4—H4	120.2	H14A—C14—H14B	109.5
C4—C5—C6	121.5 (4)	C13—C14—H14C	109.5
C4—C5—H5	119.3	H14A—C14—H14C	109.5
C6—C5—H5	119.3	H14B—C14—H14C	109.5
C1—C6—C5	118.0 (4)	C6—C15—H15A	109.5
C1—C6—C15	121.8 (4)	C6—C15—H15B	109.5
C5—C6—C15	120.1 (4)	H15A—C15—H15B	109.5
C12—C7—C8	119.0 (4)	C6—C15—H15C	109.5
C12—C7—N2	116.6 (3)	H15A—C15—H15C	109.5
C8—C7—N2	124.4 (3)	H15B—C15—H15C	109.5
C9—C8—C7	120.5 (4)	O1—C10—C9	118.2 (3)
C9—C8—H8	119.7	O1—C10—C11	121.1 (4)
C7—C8—H8	119.7	C9—C10—C11	120.7 (3)
C8—C9—C10	120.2 (4)	N2—N1—C3	113.8 (3)
C8—C9—H9	119.9	N1—N2—C7	113.7 (3)
C10—C9—H9	119.9	C10—O1—H16	109.5
			
C6—C1—C2—C3	1.3 (5)	C13—C11—C12—C7	−178.9 (3)
C1—C2—C3—C4	−1.4 (5)	C12—C11—C13—O2	−176.3 (4)
C1—C2—C3—N1	177.9 (4)	C10—C11—C13—O2	4.0 (5)
C2—C3—C4—C5	1.1 (5)	C12—C11—C13—C14	3.6 (4)
N1—C3—C4—C5	−178.2 (4)	C10—C11—C13—C14	−176.1 (3)
C3—C4—C5—C6	−0.6 (5)	C8—C9—C10—O1	−179.5 (4)
C2—C1—C6—C5	−0.8 (5)	C8—C9—C10—C11	1.8 (5)
C2—C1—C6—C15	180.0 (4)	C12—C11—C10—O1	178.9 (3)
C4—C5—C6—C1	0.5 (5)	C13—C11—C10—O1	−1.4 (5)
C4—C5—C6—C15	179.7 (4)	C12—C11—C10—C9	−2.5 (5)
C12—C7—C8—C9	−2.3 (5)	C13—C11—C10—C9	177.2 (3)
N2—C7—C8—C9	177.4 (4)	C4—C3—N1—N2	−2.4 (4)
C7—C8—C9—C10	0.7 (5)	C2—C3—N1—N2	178.4 (3)
C8—C7—C12—C11	1.6 (5)	C3—N1—N2—C7	179.7 (3)
N2—C7—C12—C11	−178.2 (3)	C12—C7—N2—N1	176.5 (3)
C10—C11—C12—C7	0.8 (5)	C8—C7—N2—N1	−3.2 (4)

### Hydrogen-bond geometry (Å, °)

**Table:**

*D*—H···*A*	*D*—H	H···*A*	*D*···*A*	*D*—H···*A*
O1—H16···O2	0.82	1.80	2.533 (4)	147
C14—H14C···O1^i^	0.96	2.53	3.318 (4)	139

Symmetry codes: (i) *x*+1, *y*, *z*.

## References

[bb1] Bahatti, H. S. & Seshadri, S. (2004). *Coloration Technol* **120**, 151–155.

[bb2] Catino, S. C. & Farris, R. E. (1985). *Concise Encyclopedia of Chemical Technology*, pp. 142–144. New York: John Wiley and Sons.

[bb3] El-Ghamry, H., Issa, R., El-Baradie, K., Isagai, K., Masaoka, S. & Sakai, K. (2008). *Acta Cryst.* E**64**, o1673–o1674. 10.1107/S1600536808024239 PMC2960714 21201664

[bb4] Fadda, A. A., Etmen, H. A., Amer, F. A., Barghout, M. & Mohammed, K. S. (1994). *J. Chem. Technol. Biotechnol.* **61**, 343–349.

[bb5] Farrugia, L. J. (1997). *J. Appl. Cryst.* **30**, 565.

[bb6] Farrugia, L. J. (1999). *J. Appl. Cryst.* **32**, 837–838.

[bb7] Klaus, H. (2003). *Industrial Dyes, Chemistry, Properties, Applications*, pp. 20–35. New York: Wiley–VCH.

[bb8] Petek, H., Erşahin, F., Albayrak, Ç., Ağar, E. & Şenel, İ. (2006). *Acta Cryst.* E**62**, o5874–o5875.

[bb9] Sheldrick, G. M. (2008). *Acta Cryst.* A**64**, 112–122. 10.1107/S0108767307043930 18156677

[bb10] Spek, A. L. (2009). *Acta Cryst.* D**65**, 148–155. 10.1107/S090744490804362X PMC2631630 19171970

[bb11] Stoe & Cie (2002). *X-AREA* and *X-RED* Stoe & Cie, Darmstadt, Germany.

[bb12] Taniike, K., Matsumoto, T., Sato, T., Ozaki, Y., Nakashima, K. & Iriyama, K. (1996). *J. Phys. Chem.* **100**, 15508–15516.

[bb13] Zollinger, H. (2003). *Color Chemistry*, 3rd revised ed. New York: Wiley-VCH.

